# Heavy Metal Levels and Cancer Risk Assessments of the Commercial Denis, *Sparus aurata* Collected from Bardawil Lake and Private Fish Farm Waters as a Cultured Source, Egypt

**DOI:** 10.1007/s12011-023-03880-0

**Published:** 2023-10-04

**Authors:** Mahmoud Mahrous M. Abbas

**Affiliations:** Marine Biology Branch, Zoology Department, Faculty of Science, Al-Azhar University, Cairo, Egypt

**Keywords:** Cancer hazard, Essential metals, ICP, Frying, Grilling, Microwave, Fish consumption

## Abstract

Heavy metal pollution of natural and cultivated habitats may be caused by agricultural, industrial, and human activities. Fish living in these habitats easily accumulate metals in their organs; for food safety and human health, the heavy metals in fish flesh are of major concern due to the harmful nature of these pollutants even in low quantities. In this study, metals (Iron, Fe; Lead, Pb; Cadmium, Cd; Nickel, Ni; Copper, Cu and Zinc, Zn) in the muscle, liver, intestine, and gill of gilthead seabream (*Sparus aurata*, Denis is local name) were monitored to determine the contamination levels and to investigate the protective impact of cooking methods on the reduction or mitigation of metal levels. Although the Denis samples exhibited relatively low Pb and Cd levels, most fish samples had elevated levels of Fe and Zn. The examined metals accumulated at the highest level in the liver and gills compared to the other organs. Results showed that cooking methods had a considerable effect on concentrations of metals. However, the levels of metal in *S. aurata* from various sources were reduced significantly (*P < 0.05*) by frying, microwave, and grilling cooking, which was ordered in the following sequence as microwave cooking < grilling < frying. The consumption of Denis fish from different sources (wild and cultured) has no negative effects on health, according to a study of health hazards based on indices of carcinogenic and non-carcinogenic. The metal results indicated that different fish sources (wild and cultured) could be acceptable for human consumption. Data hypothesized a positive impact of awareness among the native community.

**Supplementary Information:**

The online version contains supplementary material available at 10.1007/s12011-023-03880-0.

## Introduction

Metal pollution of aquatic habitats is a major environmental concern worldwide, especially in Egypt. Heavy metals are present in the aquatic environment in a variety of ways, including through untreated or insufficiently treated agricultural, domestic, and industrial effluent [1]. Metals are ingested by aquatic organisms in low concentration through water uptake and in higher concentration through biomagnification of prey; however, consumers can ingest metals through the food chain, which can have acute and long-term health effects [2]. Among aquatic species, the Denis, *Sparus aurata* receives HMs from the sediments and water in which it lives. According to Hadj Taieb et al. [3], it is opportunistic and carnivorous, which allows it to accumulate metals through the biomagnification process (via the food chain). The *S. aurata*, is an economically important demersal species inhabiting Egypt’s Mediterranean region and is one of the most important species in Egyptian marine aquaculture [4]. The major Egyptian fisheries resource for seabream is the Bardawil Lake, which is a shallow body of water with high salinity and is regarded as one of the most important sources of Egyptian fisheries [5]. Metal accumulation in Denis tissues has been observed in various scientific studies [6–8].

Heavy metals, including Fe, Cu, Co, Ni, Mn, and Zn, are necessary for biological life; but become poisonous at higher concentrations [9]. However, Mercury, Cadmium, Arsenic, and Lead are toxic and, even at low concentrations, can be hazardous [10]. According to the classifications of heavy metals, consuming them in low or high quantities could pose a serious risk. Ingestion of toxic metals at low levels over an extended period can be extremely hazardous to human health. Additionally, ingesting significant essential metal levels may be hazardous to human health. [11]. Depending on the nutritional habits, seafood might be prepared in various ways using different cooking strategies such as boiling, frying, baking, and grilling [12]. Different cooking strategies can affect the heavy metal content of fish [13]. Consequently, it is important to determine their concentrations in raw and cooked Denis fish in order to evaluate the possible risks of consumption for humans. Therefore, the risks to human health from metal pollution can be reduced when fish consumers are aware of the most effective cooking strategies to reduce metal pollution.

This study aims to evaluate some trace elements such as Fe, Pb, Cd, Ni, Cu, and Zn levels in the muscle, liver, intestine, and gills of both cultured and wild Denis fish (*S. aurata*) to calculate the contamination level and investigate the protective effect of cooking strategies (frying, microwave cooking, and grilling) on the mitigation or reduction of metal levels. It also aims to identify the health risks to fish consumers and to ensure public health safety by raising awareness of possible health hazards related to fish intake.

## Material and Methods

### Collection of Denis Fish Samples

**Fig. 1:**
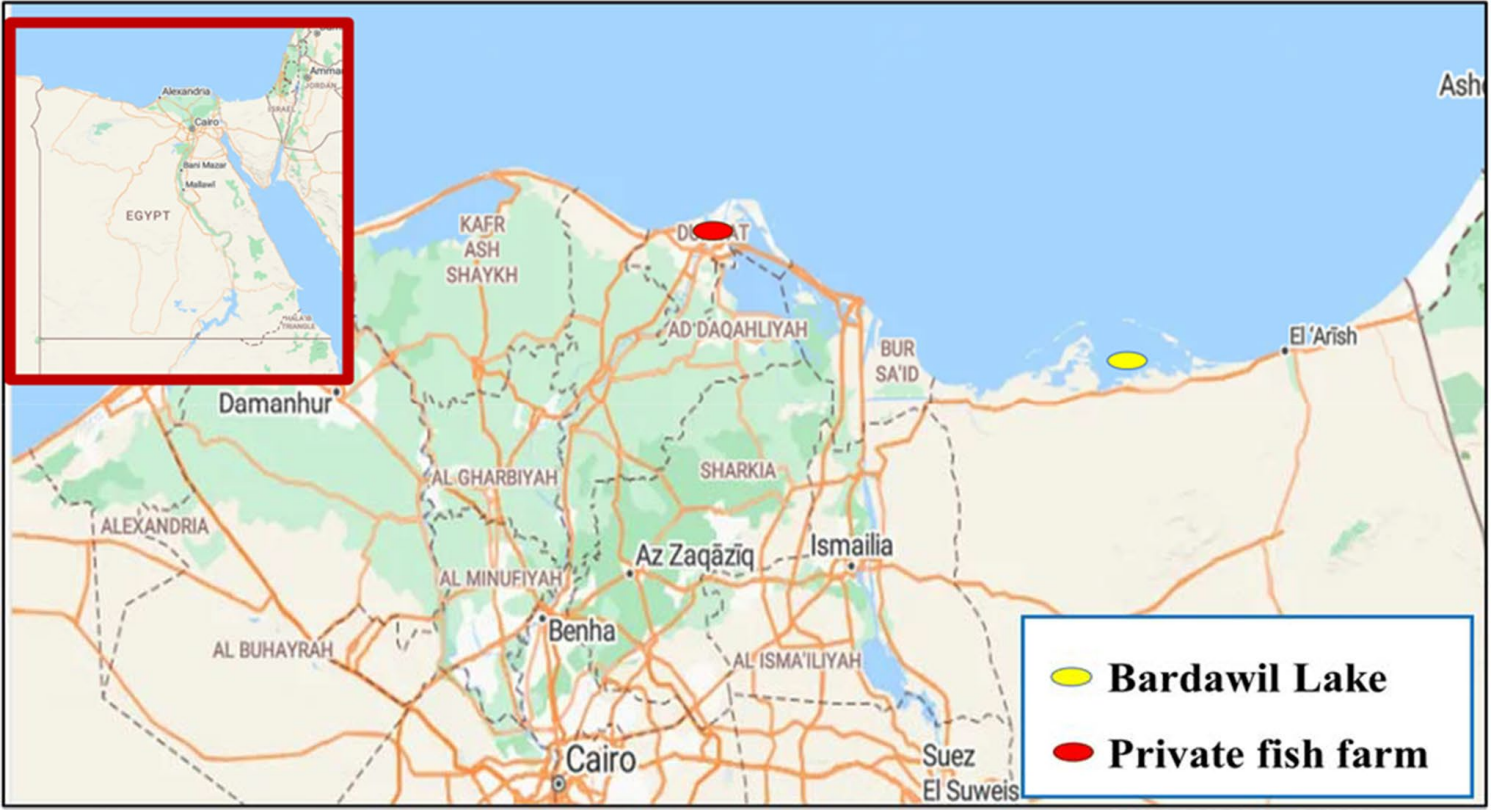
Location of the sampling of Denis fish sources in the study areas.

Eighty samples of Denis fish were collected by fishermen from two separate locations in Egypt: the first collection was from the Bardawil Lake (wild source), located at latitude of 31° 11′ 25.74″ N and longitude of 33° 09′ 44.03″ E, while the second one was from private fish farm waters (cultured source) situated in Ezbet Elborg, Domietta province, at latitude of 31° 24′ 59.33″ N and longitude of 31° 48′ 47.95″ E, both sources supplied saline-water from Mediterranean sea. Sampling was bought by local fishermen between July, to October 2022 (Fig. 1). The Denis samples were stored in an icebox after collection and transported to the marine biology laboratory for further analysis.

### Cooking Methods of Denis Fish Muscles

The samples of Denis fish were washed, cleaned, and filleted in the laboratory after being measured for weight and length. Denis fish weights and lengths were 470±23 g and 25.52 ± 0.88 cm, respectively for the cultured source and 393.02 ± 44.40 g and 23.79 ± 1.19 cm, respectively for the wild source. Filleted Denis fish were divided into four groups (10 samples from each group). The first group was uncooked fish used as references (raw), the second group was cooked through frying (Fish was fried in fresh sunflower oil for 8 min), the third group was cooked through microwave, and the fourth group was cooked through grilling (Fish was grilled in oven for 20 min).

### Heavy Metal Level Measurements

#### The Digestion of Denis Fish Samples

The Denis fish organs (raw; muscle, liver, intestine, and gill) and cooked fish (grilling, microwave cooking, and frying) samples were investigated for measurements of heavy metal level (HML). The samples (raw and cooked) were dried at 105°C for 24 h in the lab oven. About 0.5 g of dried samples were placed in 50 mL digestion vessels with ultrapure HNO_3_ (65%, 5 mL), and H_2_O_2_ (30%, 1 mL) was added. The mixture was warmed until completely digested on the hot plate. The digested samples were allowed to cool at room temperature, moved to volumetric flasks, and then mixed with HNO3 (1%), resulting in a final volume of 25 mL. The diluted solutions were then tested [14].

#### Analysis of HML in Denis Fish Organs

Levels of HM were detected in diluted solutions of Denis organs. An inductively coupled plasma optical emission spectrophotometer (Perkin Elmer, ICP-OES, 4300 DV, Shelton, USA) was employed to quantify the HML in the serial dilutions (specimens, *n*=5). The specimens treated with four calibration standards were made up of a stock solution (1 μg l^−1^) of each HM mixed in 5% (v/v) nitric acid at levels of 0, 50, 100, 200, 400 μg/L to calculate the calibration plot to determine the level of each HM in the digested mixtures. The quality control (QC) sample was checked every ten samples to ensure that both the instrument drift and calibration curve were within tolerable limits. To determine the strategy detection limit, duplicate blank specimens from each analytical group were performed in a randomized order. The calculated correlation coefficient (*R*^2^) for all calibration curves of the metals analyzed was from 0.992 to 0.999. The validation parameters of the analytical method are given in Table 1S. The recovery percentage varied from 95.36 to 98.65%. On a dry weight basis (dw-b), the contents of metals in the fish samples were assessed in μg/g dw-b [2].

### Environmental Hazard Assessment

The levels of metal pollution in aquatic species are estimated using a variety of indices [15]. In this study, the contamination status of HM in the organs of wild and cultured Denis fish was assessed using several frequently used index values, including the contamination factor (CF-HML) and the pollution index (MPI-HML) to evaluate the contamination degree of HML in Denis fish captured from different sources.

### Contamination Factor (CF-HML)

Using metal levels in Denis fish samples, the contamination factor (CF-HML) for metals was derived as the following equation:

$$\mathrm{CF}-\mathrm{HML}\;=\;{\mathrm C}_{\mathrm{HML}}/{\mathrm C}_{\mathrm{BL}}$$

where C_HML_ stands for the HML Denis fish samples (μg/g dw-b), and C_BL_ stands for background level of metals (Pb, Fe, Cd, Cu, Ni, and Zn). The values of CF-HML ≤ 1 denote a minimal limit of contamination, 1< CF-HML ≤ 2 is denoted as a low contamination degree, 2 < CF-HML ≤ 3 is moderate contamination, and CF-HML > 3 represented by a high degree of contamination [15].

### Pollution Index (MPI-HML)

The pollution index (MPI-HML) is an integrated approach to assess heavy metal pollution. This equation was used to estimate the MPI-HML [15]:

$$\mathrm{MPI}-\mathrm{HML}=\left({\mathrm{HML}}_1\;\times\;\;{\mathrm{HML}}_2\;\times\;{\mathrm{HML}}_3\;\times\cdot\cdot\cdot\cdot\cdot\cdot\times\;{\mathrm{HML}}_{\mathrm x}\right)^{1/\mathrm n}$$

where HML_1_ is the first metal level, HML_2_ is the second metal level, HML_3_ is the third metal level, n is the number of examined metals and HML_x_ is the x^th^ metal level (μg/g dw-b) in the Denis fish organs. The contamination level is safe degree when the MPI-HML value is less than 1, the MPI-HML is between 1.0 and 2.0, conditions are categorized as slightly contaminated, 2.0 to 3.0, moderately to severely contaminated, 3.0 to 5.0, severely contaminated, and > 10 heavily contaminated.

### Health Hazard Assessment

We employed a technique established by the USEPA [16] to evaluate the risk to human health of HML consumed by ingestion of the muscles of the investigated fish. The estimated daily intake (EDI**-** HML**)**, non-carcinogenic and carcinogenic indexes of HML were all performed by detecting the levels of HM in the raw muscles, fried, microwaved cooking, and grilled samples.

### Estimated Daily Intake (EDI)

The EDI**-**HML (the daily average ingestion of a specific metal during the lifespan) was used to calculate the exposure dose caused by direct human consumption of some metals observed in edible organs. The EDI**-**HML was calculated using the following formula and represented as mg/kg/day [17].

$$\mathrm{EDI}-\mathrm{HML}\;=\;\left(\mathrm{EP}\times\mathrm{IR}\times\mathrm C-\mathrm{HML}\times\mathrm{ER}/\mathrm{BW}\times\mathrm{AT}\right)\;\times10^{-3}$$

where the EP refers to the lifespan of exposure time, which is estimated to be 70 years old; the IR needs to account again for the daily ingestion of fish intake was calculated as kilograms per day, or 41 g per day for adults and 27 g per day for children. C**-**HML stands for the metal levels in raw muscles, fried, microwaved cooking, and grilled samples (μg/g wet wt.), Fish wet weight was converted to dry weight using a conversion coefficient of 4.8 [18]; ER means standing for exposure rate (365 days year^-1^); BW refers to the body weight, which was previously defined as 70 per kg in adults and 30 per kg children; The average lifetime is AT (70 years × 365 days per year).

### Non-carcinogenic Index

#### Target hazard quotient or THQ-HML

The THQ, a non-cancer evaluation of harmful health effects associated with ingesting certain HML pollutants in edible fish flesh, was established to assess human risk. The ratio of EDI-HML (average daily dosage) to the oral reference dose (mg/kg/day, ORD-HML) was used to calculate THQ-HML as:

$$\mathrm{THQ}-\mathrm{HML}\;=\;\mathrm{EDI}-\mathrm{HML}\;/\mathrm{ORD}-\mathrm{HMs}$$

According to recommendations made by the USEPA in 2018 for Pb, Cu, Cd, Ni, Fe, and Zn, the ORD-HML should be 0.00357, 0.04, 0.001, 0.02, and 0.3 mg/kg/day, respectively [16].

#### Hazard index (**HI**-HML)

The HI-HML is another mathematical formula that, according to Cui et al. [19], reflects the impact of non-carcinogenic risks by the sum of the THQ-HML values for the metals under study:

$$\mathrm{HI}-\mathrm{HML}\;=\;{\textstyle\sum_{}}\;\mathrm{THQ}-\mathrm{HML}\;\left(\mathrm{metals}\right)$$

#### Carcinogenic index (CI-HML)

The carcinogenic index (CI-HML) of heavy metal exposure lifetime was established as the incremental risk of an individual acquiring cancer depending on the carcinogenic slope factor (CSF-HML for Ni, Cd, and Pb were 0.00084, 6.3, and 0.0042 mg/kg/day, respectively). This equation was applied to calculate the CI-HML [20]:

$$\mathrm{CI}-\mathrm{HML}=\;\mathrm{EDI}-\mathrm{HML}\;\times\mathrm{CSF}\cdot-\mathrm{HML}$$

### Statistical Analysis

The SPSS, a statistical program (Version 22; software, USA), was used to perform statistical analyses. To determine a normal distribution and homogeneity of variance, Levene’s test was applied. To determine the statistically significant differences between the impacts of different cooking strategies on the level of metals, the results were statistically evaluated using analysis of variance (one-way ANOVA), and Post hoc Tukey analyses were performed when differences occurred. Additionally, to investigate the statistical differences between wild and cultured sources of each metal in Denis fish, the independent-sample *T*-test was employed. However, the correlations between the metal levels in samples of wild and cultivated Denis fish were assessed using Pearson's correlation coefficient. The statistics are presented in tables as means±standard deviation. Statistical significance, however, was represented at *p < 0.05*.

## Results and discussion

### HML in Organs of Denis Fish

Some HMLs can be found in the environment naturally. For example, essential HMLs such as Zn, Cu, and Fe have biological roles for aquatic species, but above certain threshold levels, they are potentially toxic to aquatic biota. However, other non-essential HMLs, such as Pb, As, Hg, and Cd, have no known biological role and are often toxic even at low levels [21, 22]. The levels of Pb, Fe, Cu, Ni, Cd, and Zn in the different sources-specific Denis organs, i.e., intestine, gill, muscle, and liver, revealed that there was a significant possibility of HML in the organs of Denis fish (Table 1). Likewise, the present study mentioned that the HML in the organs of wild and cultured Denis fish (gill, intestine, muscle, and liver) showed more Iron (Fe) than any studied HML, while Cadmium (Cd) was at the lowest end, and the HML ranged in this order: Fe > Cu > Zn > Ni > Pb > Cd for the studied organs (intestine, muscle, and liver). In contrast, the HML of gills is arranged in this sequencing: Fe > Zn > Cu > Ni > Pb > Cd. The most abundant of the HML studied was Iron, while Cd showed alternating levels of accumulation in Denis fish organs. This observation is in accordance with Al-Halani et al***.*** [23] who revealed that the maximum level of HM in wild fish organs, *Dicentrarchus labrax*, occurred for Fe and the minimum level was detected for Cadmium. Moreover, The HML in the wild and cultured Denis organs exhibited the minimum values recorded for muscle. The findings of the present study also confirmed the results reported by Begum et al. [24], Liu et al. [25] Abbas et al. [26] and disagreed with Zhao et al. [27], Liu et al. [28] and Liu et al. [29] [30]**,** whom reported that the muscles of marine fish recorded the highest levels of heavy metals.

**Table 1:** HML (means±SD, μg/g, dw-b) in Denis fish organs (*Sparus aurata***)** from different sources.

Metals	Fish source	Denis fish organs				Permissible limit [31]
		Gills	Muscles	Intestine	Liver	
Fe	Wild	79.58±2.32 ^**b**^	36.21±3.54 ^**d**^	53.29±10.25 ^**c**^	86.83±10.32^**a**^	100 μg/g
	Cultured	**111.25±10.25** ^**b**^	77.88±8.52 ^**d**^	81.29±6.24 ^**c**^	**118.50±7.54** ^**a**^	
*T*-test – *p*-value		4.12 – 0.002	3.29 – 0.003	2.56 – 0.016	2.82 – 0.009	
Zn	Wild	**45.85±1.65** ^**a**^	18.20±1.02 ^**d**^	19.85±1.45 ^**c**^	22.20±1.41 ^**b**^	40 μg/g
	Cultured	**49.85±2.01** ^**a**^	20.18±0.87 ^**d**^	22.21±1.02 ^**c**^	26.20±2.54 ^**b**^	
*T*-test – *p*-value		3.78 – 0.001	4.83 -< 0.001	2.57 – 0.016	2.87 – 0.008	
Pb	Wild	1.60±0.15 ^**b**^	0.84±0.07 ^**d**^	1.38±0.37 ^**c**^	1.69±0.88 ^**a**^	2 μg/g
	Cultured	1.55±0.08 ^**b**^	0.74±0.24 ^**d**^	1.28±0.68 ^**c**^	1.59±0.42 ^**a**^	
*T*-test – *p*-value		1.57 – 0.13	1.03 – 0.23	0.801 – 0.43	1.32 – 0.19	
Cu	Wild	**39.04±3.21** ^**a**^	21.79±1.24 ^**d**^	23.44±0.89 ^**c**^	**30.39±2.14** ^**b**^	30 μg/g
	Cultured	**43.04±2.65** ^**a**^	23.77±2.02 ^**d**^	25.80±1.24 ^**c**^	**34.39±1.89** ^**b**^	
*T*-test – *p*-value		2.56 – 0.016	2.41 – 0.025	2.31 – 0.028	2.50 – 0.020	
Ni	Wild	4.07±0.47 ^**b**^	1.44±0.85 ^**d**^	1.77±0.36 ^**c**^	8.39±0.84 ^**a**^	30 μg/g
	Cultured	6.72±1.24 ^**b**^	2.09±0.64 ^**d**^	3.07±0.41 ^**c**^	12.04±0.48 ^**a**^	
*T*-test – *p*-value		2.31 – 0.03	2.55 – 0.02	2.88 – 0.01	2.41 – 0.03	
Cd	Wild	**0.60±0.05** ^**b**^	0.35±0.03 ^d^	0.41±0.01 ^c^	**0.69±0.07** ^**a**^	0.5 μg/g
	Cultured	**0.52±0.07** ^**b**^	0.25±0.02 ^d^	0.32±0.05 ^c^	**0.59±0.04** ^**a**^	
*T*-test – *p*-value		0.82 – 0.41	0.92 - 0.36	1.03 – 0.23	0.92 - 0.36	

*Between wild and cultured Denis fish, metals display or do not display significant differences (T-test, p-value). While a one-way ANOVA p<0.05, reveals that findings from the same rows and fish source (among Denis organs) having different alphabetic small letters are statistically different. However, bold values are above the allowed limit

In comparison to FAO standards, the Iron levels in the wild organs of Denis fish ranged from 36.21±3.54 to 86.83±10.32 g/g, dw-b, which were lower than the acceptable limits. In the cultured organs, however, it fluctuated between 77.88±8.52 and 118.50±7.54 μg/g, dw-b. Cultured origins of muscles and intestine were lower than the permissible limit, whereas gills and liver were higher than the acceptable limits**.** According to FAO [31]**,** the Zinc levels in the wild and cultured organs of Denis fish were below the permissible levels, except for the gills (45.85±1.65 and 49.85±2.01 μg/g, dw-b, respectively). The lowest levels of Pb in the organs of wild and cultured Denis fish were 0.84±0.07 and 0.74±0.24 μg/g, dw-b, respectively, and the highest levels were 1.69±0.88 and 1.59±0.42 μg/g, dw-b, respectively. All four organs of the Denis fish tested for Pb levels were below the acceptable limits estimated by the FAO [31]. Copper levels in the wild and cultured organs of Denis fish ranged between 21.79±1.24 to 39.04±3.21 μg/g, dw-b in the former origin and 23.77±2.02 to 43.04±2.65 μg/g, dw-b in the second one. Cu levels in the muscles and intestine were lower than the permissible limit, whereas the gills and liver were higher than the acceptable limits estimated by the FAO [31]**.** The minimal levels of Nickel in the organs of wild and cultured Denis fish were 1.44±0.85 and 2.09±0.64 μg/g, dw-b, respectively and the maximal levels were 8.39±0.84 and 12.04±0.48 μg/g, dw-b, respectively. All four organs of Denis fish from wild and cultured origins tested for Ni levels were below the acceptable limits estimated by the FAO [31]**.** Cadmium levels in the wild and cultured organs of Denis fish ranged between 0.35±0.03 to 0.69±0.07 μg/g, dw-b in the former origin and 0.25±0.02 to 0.59±0.04 μg/g, dw-b in the second one. Cd levels in the muscles and intestine were lower than the permissible limit, whereas gills and liver were higher than the acceptable limits estimated by the FAO [31]**.**

In most cases, the essential metals exhibited higher levels in cultured Denis organs than in wild Denis organs; this may be attributed to the fact that these metals are required for different biological activities and thus supplied into fish diets [22, 26, 32]**,** suggesting that cultured fish have higher levels [33–35].

This observation agrees with Yipel et al. [36]**,** who reported that the wild fish, *Sparus aurata,* accumulate less Fe and Zn than the cultured ones. However, the level of toxic metals in wild Dines fish organs was significantly lower than in cultured fish organs (*P < 0.05*), which may be attributed to wild fish surviving over several years compared to cultured fish, which are captured within six months. Wild Denis organs can accumulate pollutants with prolonged biological lifespans, notably both Cadmium and Lead, over a longer lifetime compared to cultured Denis organs. According to Chatta et al. [37], the Lead and Cadmium absorbed in the cultured *Labeo rohita* and *Cirrhinus mrigala* were lower than in the wild ones.

The cadmium levels exhibited the lowest values in the organs of Denis fish (*Sparus aurata***)** from both studied sources. Cadmium is extremely harmful due to its extremely potential toxicity even at low levels, its persistence in the environment, and its proclivity for bioaccumulation in aquatic biota. In the aquatic biota, Cd is not digested by the body, and it accumulates in the soft tissues and becomes poisonous, and as a direct consequence of their bioaccumulation, the food chain has become contaminated, affecting the entire ecological activity. Nowadays, global attention becomes more critical for African countries such as Egypt, where the pressure from exploding the population requires a lot of food supply [38–42]. Additionally, Perera et al. [43] stated that natural and anthropogenic activities can be identified as an important source of Cd to the biosphere. Natural emissions are mainly from the mobilization of naturally occurring Cd from the earth’s crust and mantle, e.g. volcanic eruptions and weathering of rocks. Anthropogenic sources are mainly from the mobilization of Cd impurities in raw materials (e.g., phosphate minerals, fossil fuels) and emissions from the manufacturing, use, disposal, recycling, reclamation, or incineration of products intentionally.

### Environmental Hazards Estimation

**Fig. 2:**
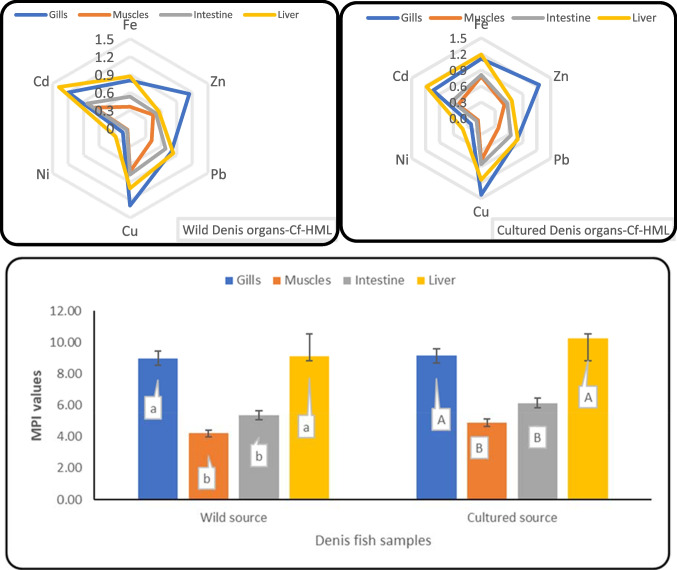
**Cf-HML, and MPI-MHL (averages±SD)** based on HML in the organs of Denis fish (*Sparus aurata*) from different sources; a one-way ANOVA, reveals that results from the same fish source having different alphabetic small letters (Wild Denis source) and capital letters (Cultured Denis source) are significantly different (*p*<0.05).

The contamination factor (Cf-HML), and metal pollution index (MPI-HML) were all applied to evaluate the contamination degree of HML in different organs of fish [15]. The evaluated Cf-HML values for the studied HML in wild Denis fish organs ranged from 0.05 to 1.38 μg g^−1^, and from 0.07 to 1.43 μg g^−1^ in cultured Denis fish organs (Fig. 2). The Cf-Pb and Cf-Cd values were higher in the wild fish organs compared with cultured Denis organs suggesting that their pollution level was relatively increased in organs of cultured Denis, in accordance with previous studies, Pb and Cd pose significant potential ecological risks [44, 45]. Cf-HML levels in Denis organs dropped in the following order: gills > liver > intestine > muscles for Cf-Fe, Cf-Pb, Cf-Ni, and Cf-Cd, whereas gills > liver > intestine > muscles for Cf-Zn and Cf-Cu. Moreover, the computed values of CF-HML in the Denis fish showed a low level of contamination (Cf-HML<1) detected in the intestines and muscles of two sources. Contrarily, Cf-Fe values in the gills and liver of cultured Denis fish had moderate contamination (1 ≥ CF ≤3), and Cf-Zn, Cf-Cu, and Cf-Cd in the gills and liver from different sources had moderate contamination. Additionally, Cf-HML values for Fe, Zn, Cu, and Ni were lower in the wild organs compared with cultured Denis organs, while they were higher for Pb and Cd. This indicates that cultured Denis may be highly contaminated with the essential metals (Fe, Zn, Cu, and Ni), while highly contaminated wild fish organs with non-essential metals were also found (Pb and Cd). The estimated values for MPI-HML in cultured sources of Denis organs were higher than the wild ones, suggesting a high pollution degree in cultured organs. However, the contamination degree based on HML in Denis fish organs can be classified as follows: Gills > liver > intestine > muscle for wild organs, while liver > gills > intestine > muscle for cultured organs, according to the estimated data resulting from MPI-HML (Fig. 2). Therefore, the high values in pollution indices of cultured Denis compared to wild sources raise concern for consumers' health due to metal contamination. Hence, it is possible to determine the potential impact on the reduction of HML by using cooking methods.

### Pearson Correlation of HML in the Denis Fish Origin

Pearson correlation (*r*) was evaluated to calculate if some of these metals were interrelated with each other based on HML in the wild and cultured Denis organs (Table 2S). The positive correlation between the MHL indicates a similar input source of metal, while the negative correlation indicates a different source. In this study, Pearson correlation analysis based on HML in organs of wild Denis fish showed a significant positive correlation between Fe-Ni, Fe-Cd, Pb-Ni, Cu-Ni, and Cu-Cd, whereas a significant negative relationship was revealed between Cd-Ni, Pb-Cd, and Pb-Cu. In the cultured Denis fish, however, correlation analysis showed a significant positive correlation between essential metals with each other (Fe, Ni, Cu, and Zn), while a significant negative correlation was shown between Cd and the studied HML, except Zn; Lead-Copper, and Lead-Nickel.

### Effect of Cooking Strategies on HML

Cooking the wild and cultured Denis muscle identifies any changes in HML, providing a significantly (*P < 0.05*) accurate representation of the potential human consumption of HML. Every day, humans cook using various cooking strategies of their choice, and fish is almost never consumed uncooked, especially in Egypt. This research chose frying, grilling, and microwave cooking as examples of cooking strategies on the HML in the muscle of Denis fish (**Table** **2**). Reduction percentages of HML in cooked Denis fish from different sources were represented in Table 1S. The grilling of wild and cultured Denis samples resulted in the reduction of Lead (56.60 and 63.87%, respectively), Cadmium (53.46 and 55.96%, respectively), Nickel (45.13 and 31.10%, respectively), Iron (11.74 and 5.46%, respectively), Copper (7.57 and 6.94%, respectively), and Zinc (23.36 and 21.06%, respectively). In accordance with our findings, Abd-Elghany et al. [46] revealed that the levels of Lead (10%) and Arsenic (50 %) in raw shrimps were lowered by 10 % and 24 %, respectively in grilled shrimp, and by 27 % and 36 %, respectively in crabs caught in the Mediterranean Sea and cooked on the grill. Additionally, Ersoy et al. [47] found lower levels of Cr, As, and Pb and higher levels of Nickel in grilled-farmed seabass caught in Turkey. Abu-Raya et al. [48] mentioned that the Copper, Lead, and Cd levels increased after grilling Bolti fish compared to raw samples, while the Zn level declined. In contrast to our observations, an increment in the levels of Hg, Cu, Se, Mn, Zn, As, and Sr by 10–55% and a lowering in Iron and Cadmium levels by 27–66% after grilling techniques for cultivated Meagre fish were observed in Portugal [49]**.** According to Kalogeropoulos et al. [50], grilled anchovy have higher levels of Cd, Zn, Hg, Fe, Pb, Ni, Cu, and Cr than their raw flesh.

**Table 2:** HML (means±SD, μg/g, dw-b) in raw and cooked Denis fish samples (*Sparus aurata***)** from different sources.

		Raw	Cooked samples		
			Microwaved	Grilled	Fried
**Fe**	Wild	36.21±3.54^a^	11.57±1.24 ^d^	23.89±3.05 ^c^	29.89±2.32^b^
	Cultured	77.88±8.52 ^a^	16.28±3.65 ^d^	47.08±3.44 ^c^	62.08±4.55 ^b^
**Zn**	Wild	18.20±1.02 ^a^	8.72±1.02 ^d^	13.46±1.33 ^c^	15.04±1.65 ^b^
	Cultured	20.18±0.87 ^a^	3.86±0.99 ^d^	8.33±0.88 ^c^	12.28±1.05 ^b^
**Pb**	Wild	0.84±0.07 ^a^	0.11±0.06 ^d^	0.24±0.06 ^c^	0.39±0.04 ^b^
	Cultured	0.74±0.24 ^a^	0.09±0.01 ^d^	0.20±0.05 ^c^	0.35±0.07 ^b^
**Cu**	Wild	21.79±1.24 ^a^	5.46±0.67 ^d^	7.81±0.87 ^c^	12.84±1.33 ^b^
	Cultured	23.77±2.02 ^a^	10.53±1.65 ^d^	15.79±1.57 ^c^	18.45±1.66 ^b^
Ni	Wild	1.44±0.85 ^a^	0.36±0.05 ^d^	0.90±0.07 ^c^	1.08±0.75 ^b^
	Cultured	2.09±0.64 ^a^	0.14±0.05 ^d^	1.12±0.57 ^c^	1.44±0.65 ^b^
**Cd**	Wild	0.35±0.03 ^a^	0.18±0.04 ^d^	0.27±0.04 ^c^	0.30±0.02 ^b^
	Cultured	0.25±0.02 ^a^	0.12±0.01 ^d^	0.20±0.02 ^c^	0.22±0.03 ^b^

*Note: One-way ANOVA, reveals that findings from the same rows and fish source having different alphabetic small letters are statistically different (*p*<0.05).

Frying of wild and cultured Denis samples exhibited a significant minimization of Lead (45.28 and 50.97%, respectively), Cadmium (12.56 and 17.51%, respectively), Nickel (24.99 and 17.22%, respectively), Iron (9.28 and 4.31%, respectively), Copper (6.24 and 5.72%, respectively), and Zinc (18.46 and 16.65%, respectively). The minimization of HML after frying may be attributed to moisture loss and fat increase during the frying strategy. The outcomes are consistent with the investigation on seabass conducted by Hosseini et al. [51], who determined that the frying method reduced Iron and Zinc levels, whereas increasing Manganese and Copper levels, and Arisekar et al. [52] who calculated that the levels of HML in *Penaeus vannamei* varied mainly due to moisture loss and uptake of oil. Pb and Ni levels were significantly lower (*P < 0.05*) than in the muscle of *Thunnus tonggol,* longtail tuna, after the frying strategy [53]. The levels of Hg, Zn, Fe, Cu, Cd, Pb, Cr, and Ni in hake, anchovy, bogue, picarel, sand smelt, sardine, stripped mullet, Mediterranean mussel, squid, and shrimp were significantly higher after domestic pan-frying [50]. Ersoy et al. [47] recorded that the Lead levels in raw and fried fish were 0.278, and 0.277 mg kg^−1^, respectively. The minimization of Fe, Cd, Ni, Mn, Zn, As, Se, and Cu after shrimp frying was evaluated as 96.4, 49.1, 67.3, 19.8, 95.3, 99.2, 89.6, and 75.3 %, respectively. Copper and Zinc levels declined after frying in Rainbow trout muscles [54]. A minimization trend in Cadmium and Zinc was observed in sardines fishes [55]. However, Abu-Raya et al 2007 revealed that Copper, and Cadmium levels after frying of Bolti fish were higher compared to raw samples, while the Zinc and Lead levels declined. However, Lead levels were not significantly lowered after the frying of sea bass [47].

The microwave cooking of wild and cultured Denis muscles exhibited a significant reduction in HML levels by 81.74, 92.51% for Pb; 66.14, 77.27% for Cd; 65.96, 45.45% for Ni; 27.48, 12.78% for Fe; 18.13, 16.62% for Cu and 43.69, 36.10% for Zn. Also, the higher in HML minimization observed after microwave cooking than the processes of grilling and frying, Arisekar et al. [52] reported that after microwave cooking, Fe, Cr, Ni, Zn, and Cu levels in shrimp tissue decreased by 25.7, 47.5, 11.5, 32.5, and 57.7 percent, respectively. The reductions in Zn, Fe, Cu, Pb, and Cr levels were observed after the microwave strategy of rainbow trout [56], sea bass [51] and catfish [47]. Previous studies mentioned that the higher levels of HML after the microwave strategy are related to oil decline/uptake and water loss [55, 57]. This minimization in HML may be due to the denaturation of proteins. Microwave heating consistently results in a higher level of protein denature than traditional methods of cooking [58], and it also leaches away proteins that bind Iron, Chromium, and Copper [59]. However, microwaved cooking of catfish resulted in no changes in the Lead level [56].

According to our findings, the HML in the muscles of the Denis fish was significantly (*P < 0.05*) affected by the cooking techniques. Cooking techniques, including microwaving, grilling, and frying, showed a significant (*P*< 0.05) reduction in the studied HML levels (Ni, Zn, Pb, Cu, Cd, and Fe) observed in the muscle of wild and cultivated Denis. HML was found in raw and cooked samples in the following order: raw > fried > grilled > microwaved samples. These outcomes might be the result of changes in moisture and fat content that happened during microwave, grilling, and frying cooking. The reduction of HML in fish cooked to different temperatures varies according to the physico-chemical features of HML and their chemical variation, the sulfhydryl link between the protein and the HML, the species, size, and the cooking variables, such as the duration, temperature, and cooking condition [50, 57]. It would be significantly (*P < 0.05*) more accurate to assess the potential health effects of ingestion by tracking changes in HML in cooked Denis fish.

### Health Hazards Estimation

**Fig. 3:**
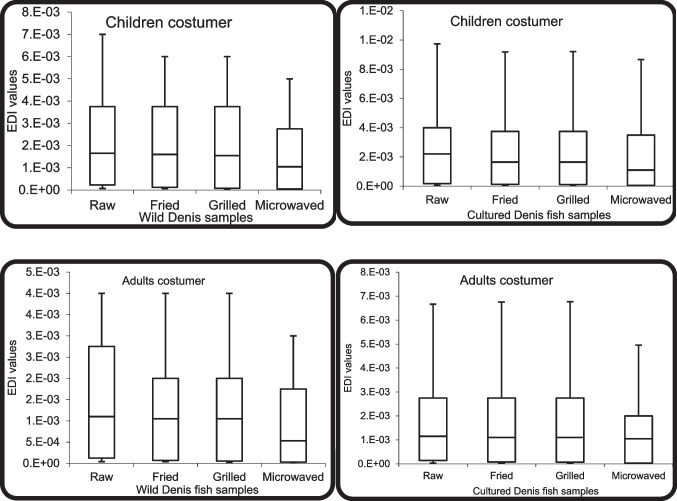
EDI-HML values (mg/kg/day) of studied heavy metals through different ways of consumptions for adults and children in the raw and cooked Denis fish samples.

Consumers’ daily exposure to HML through eating foods high in HML was employed to avoid any detrimental effects on humans during their lifespan [60]. EDI (mg kg^−1^day^−1^) for HML in the raw and cooked muscles of both cultured and wild Denis fish are represented in Table 4S and Fig. 3. The EDI-HML values of Fe, Zn, Cu, Ni, Cd, and Pb for consumers (children and adults) were lower than the PTDI (permissible tolerable daily intake). The PTDI values of Fe, Zn, Cu, Ni, Cd, and Pb are 50, 70, 50, 4E-02, 3E-03, and 3E-02 mg/kg/day, respectively [61]. The recorded EDI-HML of both groups was compared to that for 70 kg of body weight [61], and it was concluded that the mean EDI values of the metals for consumers (children and adults) do not exceed the PTDI values. The EDI-HML values for the essential metals in the raw and cooked Denis muscles of cultivated origin were higher than those of wild origin, indicating that consumers (children and adults) had a higher rate of exposure when consuming the cultivated fish than those of wild origin. On the other hand, the EDI-HML values for the non-essential metals in the raw and cooked Denis muscles of cultivated origin were lower in comparison to those of wild origin, which suggested that consumers (children and adults) had a higher rate of exposure when consuming wild fish than those cultivated.

**Fig. 4:**
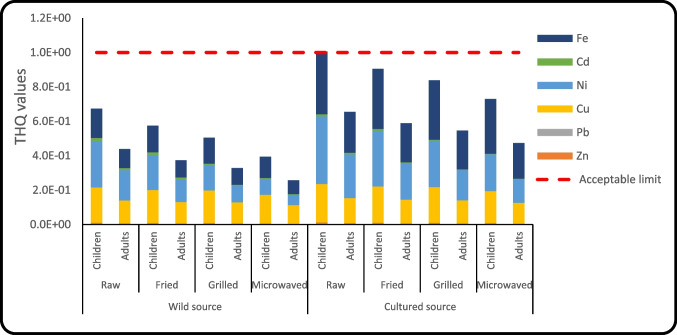
The values of THQ- HML in the raw and cooked Denis fish samples.

**Fig. 5:**
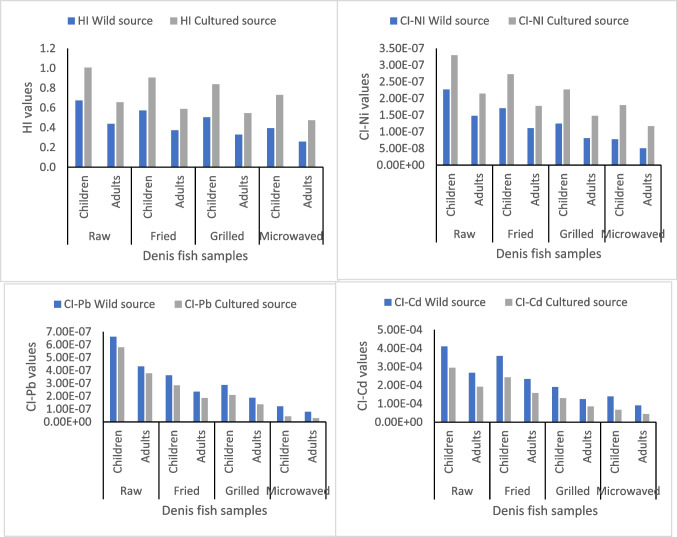
The hazard index (HI) and risk index (CI-Ni, CI-Pb and CI-Cd) in raw and cooked Denis fish samples (*Sparus aurata***)** from different sources (wild and cultured).

Target hazard quotient (THQ-HML) for Cu, Zn, Fe, Ni, Cd, and Pb in raw and cooked muscles of both cultured and wild fish are illustrated in Table 4S and Fig. 4. The allowable threshold level of THQ-HML is one [16]. The THQ-HML values determined in edible Denis fish were under 1, suggesting that eating muscles won't have any adverse health effects for consumers (children and adults) who consumed the studied Denis fish. Furthermore, the hazard index (HI-HML) values for both adults and children through consumption of the two fish sources were evaluated based on the THQ-HML values; if the HI-HML value was less than one (HI-HML ≤1), the effects on humans would be adverse, HI-HML > 1 most probably had a negative impact; and HI-HML >10 strong or chronic of acute implications, as recommended by [62]**.** The HI-HML values in raw and cooked muscles of both cultured and wild Denis fish for both consumers (children and adults) were less than one, suggesting no hazard for human consumption occurred (Table 5S and Fig. 5).

The carcinogenic index (CI-HML) values for Cd, Ni, and Pb in the raw and cooked muscles of both cultured and wild Denis fish were determined for both adult and child eaters, and the results are presented in Table 5S and Fig. 5. The CI-Pb and CI-Ni values in the raw and cooked muscles of Denis fish were lower than 1E−6 for children as well as adult consumers, meaning that the carcinogenic hazard caused by Pb and Ni was safe [63]. However, Cd poses a carcinogenic risk (CI-Cd) to adult as well as children’s consumers of Denis fish, as the values of CI-Cd were higher than the acceptable value of 1E−6 [60].

Non-carcinogenic indexes (THQ and HI) and Nickel carcinogenic index (CI-Ni) of metals for both consumers (children and adults) were higher in cultured muscles of raw and cooked Denis fish compared to wild Denis fish. However, the carcinogenic indexes of Cadmium and Lead (CI-Cd and CI-Pb) for both consumers (children and adults) were lower in cultured muscles of raw and cooked Denis fish compared to wild Denis fish. These results agree with Tahity et al. [15]**.**

## Conclusion

Cooking strategies (grilling, microwave cooking, and frying) were applied to determine whether the metal levels in cultured and wild Denis muscles may be declined to a safe level or avoided it. The levels of essential HML were a significantly decreased (*P < 0.05*) in the wild Denis organs compared to cultured ones. Non-essential HML, concentrations increased significantly (*P < 0.05*) in wild Denis organs compared to cultured ones. Moreover, the slightly high values in pollution indices of cultured Denis compared to wild sources (Bardawil Lake) raise concern for consumers' health due to metal contamination. Hence, it is possible to detect the potential impact of heavy metals by using cooking methods. Results indicated that the levels of the examined metals declined in the sequence of frying > grilling > microwaved cooking. For more accuracy in evaluating the possible risks to human health from eating both wild and cultivated Denis muscles, it was confirmed that the cooking processes gave consumers a clear view of the potential hazards and showed that there was no non-carcinogenic hazard, as well as no carcinogenic risk for Nickel and Lead, except Cadmium, which poses a carcinogenic hazard to adult and children consumers of wild and cultured Denis cooked muscles. These findings show that it is vital to apply the cooking strategies for muscles of wild and cultured Denis to minimize possible health hazards. Therefore, a long-term management strategy and biomonitoring of these HM in Lake Bardawil as Egyptian vision of 2030 and around fish farm waters are required. This will reduce the amount of pollution in the aquatic ecosystem, which represents a health risk to humans who consume polluted fish with HMs.

## Supplementary information

ESM 1

## Data Availability

The data sets in this study are available from the corresponding author upon reasonable request.
