# Supplementary material for: Heavy Metal Levels and Cancer Risk Assessments of the Commercial Denis, Sparus aurata Collected from Bardawil Lake and Private Fish Farm Waters as a Cultured Source, Egypt
Source: Biol Trace Elem Res. 2023 Oct 4;202(6):2864–77. doi: 10.1007/s12011-023-03880-0 (PMC11534982; doi:10.1007/s12011-023-03880-0)
Supplement: Supplementary file 1 — ESM 1 [file 12011_2023_3880_MOESM1_ESM.docx]

**Table 1S.** Validation parameters of the analytical method.

|  | LOD (ng g−1) | **LOQ (ng g^−1^)** | RSDr (%) | **Recovery (%)** | R^2^ |
| --- | --- | --- | --- | --- | --- |
| Fe | 1.60 | 6.28 | 1.87 | 95.36 | 0.992 |
| Ni | 0.51 | 1.64 | 2.24 | 96.35 | 0.999 |
| Cu | 0.67 | 2.01 | 1.95 | 98.65 | 0.993 |
| Zn | 2.42 | 6.50 | 2.65 | 97.65 | 0.993 |
| Cd | 0.48 | 1.58 | 1.79 | 96.35 | 0.992 |
| Pb | 0.43 | 1.13 | 1.98 | 98.32 | 0.993 |

**Table 2S.** Pearson correlation coefficients based on HML in the Denis fish (*Sparus aurata***)** from different sources.

|  | **Wild source** | | | | | |  |  |  |
| --- | --- | --- | --- | --- | --- | --- | --- | --- | --- |
|  | *Fe* | *Zn* | *Pb* | *Cu* | *Ni* | *Cd* |  |  |  |
| Fe | 1 | 0.908 | 0.535 | 0.39 | 0.692 | 0.656 |  |  |  |
| Zn | **0.908** | 1 | 0.465 | 0.208 | 0.157 | 0.491 |  | 1 | Positive correlation |
| Pb | 0.535 | 0.465 | 1 | -0.72 | 0.682 | -0.641 |  |  |  |
| Cu | 0.39 | 0.208 | **-0.72** | 1 | 0.714 | 0.709 |  | 0.5 |  |
| Ni | **0.692** | 0.157 | **0.682** | **0.714** | 1 | -0.815 |  |  |  |
| Cd | **0.656** | 0.491 | **-0.641** | **0.709** | **-0.815** | **1** |  | 0 |  |
|  | **Cultured source** | | | | | |  |  |  |
|  | *Fe* | *Zn* | *Pb* | *Cu* | *Ni* | *Cd* |  | -0.5 | Negative correlation |
| Fe | 1 | **0.743** | **0.766** | **0.86** | **0.747** | **-0.625** |  |  |  |
| Zn | **0.743** | 1 | 0.464 | **0.915** | **0.686** | -0.266 |  | -1 |  |
| Pb | **0.766** | 0.464 | 1 | **-0.803** | **-0.730** | **-0.707** |  |  |  |
| Cu | **0.860** | **0.915** | **-0.803** | 1 | **0.682** | **-0.761** |  |  |  |
| Ni | **0.747** | **0.686** | **-0.730** | **0.682** | 1 | **-0.822** |  |  |  |
| Cd | **-0.625** | -0.266 | **-0.707** | **-0.761** | **-0.822** | **1** |  |  |  |

** Bold correlation values are significant at p < 0.05 (two-tail).*

**Table 3S.** Reduction percentages of HML in cooked Denis fish from different sources RP % = ((raw-HML – cooked-HML)/Raw-HML)x 100.

|  | **RP %** | Cooked samples | | |
| --- | --- | --- | --- | --- |
|  |  | Fried | Grilled | Microwaved |
| **Fe** | wild | 9.28 | 11.74 | 27.48 |
|  | cultured | 4.31 | 5.46 | 12.78 |
| **Zn** | wild | 18.46 | 23.36 | 43.69 |
|  | cultured | 16.65 | 21.06 | 36.10 |
| **Pb** | wild | 45.28 | 56.60 | 81.74 |
|  | cultured | 50.97 | 63.87 | 92.51 |
| **Cu** | wild | 6.24 | 7.57 | 18.13 |
|  | cultured | 5.72 | 6.94 | 16.62 |
| Ni | wild | 24.99 | 45.13 | 65.96 |
|  | cultured | 17.22 | 31.10 | 45.45 |
| **Cd** | wild | 12.56 | 53.46 | 66.14 |
|  | cultured | 17.51 | 55.96 | 77.27 |

|  | | EDI-HML (mg/kg/day) | | | | | | | | THQ-HML | | | | | | | | HM  PTDI |
| --- | --- | --- | --- | --- | --- | --- | --- | --- | --- | --- | --- | --- | --- | --- | --- | --- | --- | --- |
|  |  | Raw | | Fried | | Grilled | | Microwaved | | Raw | | Fried | | Grilled | | Microwaved | |  |
|  |  | Children | Adults | Children | Adults | Children | Adults | Children | Adults | Children | Adults | Children | Adults | Children | Adults | Children | Adults |  |
| **Fe** | wild | 6.8E-03 | 4.4E-03 | 6.2E-03 | 4.0E-03 | 6.0E-03 | 3.9E-03 | 6.8E-03 | 4.4E-03 | 0.17 | 0.11 | 0.154 | 0.1 | 0.15 | 0.097 | 0.123 | 0.08 | 50 |
|  | cultured | 1.5E-02 | 9.5E-03 | 1.4E-02 | 9.1E-03 | 1.4E-02 | 9.0E-03 | 1.5E-02 | 9.5E-03 | 0.365 | 0.238 | 0.349 | 0.227 | 0.345 | 0.225 | 0.318 | 0.207 |  |
| **Zn** | wild | 3.4E-03 | 2.2E-03 | 2.8E-03 | 1.8E-03 | 2.6E-03 | 1.7E-03 | 3.4E-03 | 2.2E-03 | 0.011 | 0.007 | 0.009 | 0.006 | 0.009 | 0.006 | 0.006 | 0.004 | 70 |
|  | cultured | 3.8E-03 | 2.5E-03 | 3.2E-03 | 2.1E-03 | 3.0E-03 | 1.9E-03 | 3.8E-03 | 2.5E-03 | 0.013 | 0.008 | 0.011 | 0.007 | 0.01 | 0.006 | 0.008 | 0.005 |  |
| **Pb** | wild | 1.6E-04 | 1.0E-04 | 8.6E-05 | 5.6E-05 | 6.8E-05 | 4.4E-05 | 1.6E-04 | 1.0E-04 | 0.0002 | 0.0001 | 0.0001 | 0.00008 | 0.0001 | 0.00006 | 0.00004 | 3.00E-05 | 3E-02 |
|  | cultured | 1.4E-04 | 9.0E-05 | 6.8E-05 | 4.4E-05 | 5.0E-05 | 3.2E-05 | 1.4E-04 | 9.0E-05 | 0.0002 | 0.0001 | 0.0001 | 0.00006 | 0.00007 | 0.00005 | 0.00001 | 1.00E-05 |  |
| **Cu** | wild | 4.1E-03 | 2.7E-03 | 3.8E-03 | 2.5E-03 | 3.8E-03 | 2.5E-03 | 4.1E-03 | 2.7E-03 | 0.204 | 0.133 | 0.192 | 0.125 | 0.189 | 0.123 | 0.167 | 0.109 | 50 |
|  | cultured | 4.5E-03 | 2.9E-03 | 4.2E-03 | 2.7E-03 | 4.1E-03 | 2.7E-03 | 4.5E-03 | 2.9E-03 | 0.223 | 0.145 | 0.21 | 0.137 | 0.207 | 0.135 | 0.186 | 0.121 |  |
| Ni | wild | 2.7E-04 | 1.8E-04 | 2.0E-04 | 1.3E-04 | 1.5E-04 | 9.6E-05 | 2.7E-04 | 1.8E-04 | 0.27 | 0.176 | 0.203 | 0.132 | 0.148 | 0.096 | 0.092 | 0.06 | 4E-02 |
|  | cultured | 3.9E-04 | 2.6E-04 | 3.2E-04 | 2.1E-04 | 2.7E-04 | 1.8E-04 | 3.9E-04 | 2.6E-04 | 0.392 | 0.255 | 0.324 | 0.211 | 0.27 | 0.176 | 0.214 | 0.139 |  |
| **Cd** | wild | 6.5E-05 | 4.2E-05 | 5.7E-05 | 3.7E-05 | 3.0E-05 | 2.0E-05 | 6.5E-05 | 4.2E-05 | 0.018 | 0.012 | 0.016 | 0.01 | 0.008 | 0.006 | 0.006 | 0.004 | 3E-03 |
|  | cultured | 4.7E-05 | 3.0E-05 | 3.9E-05 | 2.5E-05 | 2.1E-05 | 1.3E-05 | 4.7E-05 | 3.0E-05 | 0.013 | 0.009 | 0.011 | 0.007 | 0.006 | 0.004 | 0.003 | 0.002 |  |

Table 4S: EDI-HML (mg/kg/day) and THQ-HML in the raw and cooked Denis fish samples (*Sparus aurata***)** from different sources (wild and cultured).

Table 5S: The hazard index (HI-HML) and carcinogenic index (CI-HML) in raw and cooked Denis fish samples (*Sparus aurata***)** from different sources (wild and cultured).

|  | | | Raw | | Fried | | Grilled | | Microwaved | |
| --- | --- | --- | --- | --- | --- | --- | --- | --- | --- | --- |
|  |  |  | Children | Adults | Children | Adults | Children | Adults | Children | Adults |
| HI-HML | | Wild source | 0.674 | 0.439 | 0.573 | 0.373 | 0.504 | 0.328 | 0.395 | 0.257 |
|  |  | Cultured source | 1.006 | 0.655 | 0.905 | 0.589 | 0.838 | 0.546 | 0.729 | 0.474 |
| CI-HML | **Pb** | Wild source | 7E-07 | 4E-07 | 4E-07 | 2E-07 | 3E-07 | 2E-07 | 1E-07 | 8E-08 |
|  |  | Cultured source | 6E-07 | 4E-07 | 3E-07 | 2E-07 | 2E-07 | 1E-07 | 4E-08 | 3E-08 |
|  | Ni | Wild source | 2E-07 | 1E-07 | 2E-07 | 1E-07 | 1E-07 | 8E-08 | 8E-08 | 5E-08 |
|  |  | Cultured source | 3E-07 | 2E-07 | 3E-07 | 2E-07 | 2E-07 | 1E-07 | 2E-07 | 1E-07 |
|  | **Cd** | Wild source | 4E-04 | 3E-04 | 4E-04 | 2E-04 | 2E-04 | 1E-04 | 1E-04 | 9E-05 |
|  |  | Cultured source | 3E-04 | 2E-04 | 2E-04 | 2E-04 | 1E-04 | 8E-05 | 7E-05 | 4E-05 |
